# Prognostic factors of all-cause mortalities in continuous ambulatory peritoneal dialysis: a cohort study

**DOI:** 10.1186/1471-2369-14-28

**Published:** 2013-01-31

**Authors:** Phisitt Vejakama, Ammarin Thakkinstian, Atiporn Ingsathit, Prateep Dhanakijcharoen, John Attia

**Affiliations:** 1 Section for Clinical Epidemiology and Biostatistics, Faculty of Medicine, Ramathibodi Hospital, Bangkok, Thailand; 2 Bundarik Hospital, Bundarik district, Ubon Ratchathani province, Thailand; 3 National Health Security Office, Bangkok, Thailand; 4 Centre for Clinical Epidemiology and Biostatistics, School of Medicine and Public Health, University of Newcastle and Hunter Medical Research Institute, Newcastle, NSW, Australia

**Keywords:** Adequacy indices, CAPD, Continuous ambulatory peritoneal dialysis, Mortality, Peritoneal small solute clearance, Prognostic factors

## Abstract

**Background:**

The role of small solute clearance on mortalities in patients with CAPD has been controversial. We therefore conducted a study with 3 years' follow up in adult patients who participated in the CAPD-first policy.

**Methods:**

There were 11,523 patients with end-stage renal disease who participated in the CAPD-first policy between 2008 and 2011. Among them, 1,177 patients were included in the retrospective cohort study. A receiver operating characteristic curve was applied to calibrate the cutoffs of tKt/V, rKt/V and tCrcl. Kaplan-Meier and Cox-regression models with time varying covariates were applied to estimate overall death rate, probability of death and prognosis, respectively.

**Results:**

The cutoffs of rKt/V and tKt/V were 0.25 and 1.75, respectively. The Cox regression suggested that the higher these clearance parameters, the lower the risks of death after adjusting for covariables. The risks of death for those above these cutoffs were 57% (HR = 0.43, 95% CI: 0.31, 0.60) and 29% (HR = 0.71, 95% CI: 0.52, 0.98) lower for rKt/V and tKt/V, respectively. Age, serum albumin, hemoglobin, systolic blood pressure, and ultra-filtration volume significantly affected the mortality outcome.

**Conclusions:**

Our study suggested that the cutoffs of 0.25 and 1.75 for rKt/V and tKt/V might be associated with mortality in CAPD patients. A minimum tKt/V of 1.75 should be targeted, but increased dialysis dosage to achieve tKt/V > 2.19 adds no further benefit. Serum albumin, hemoglobin, SBP, and UF volume are also associated with mortality. However, our study may face with selection and other unobserved confounders, so further randomized controlled trials are required to confirm these cutoffs.

## Background

The global burden of end-stage kidney disease (ESRD) is increasing rapidly [1-3] with a prevalence of 0.2% in the USA, and 0.3% in Thailand respectively [4,5]. Renal replacement therapy (i.e., renal transplantation or dialysis) is required for ESRD patients, and 2 modalities, hemo-dialysis and continuous ambulatory peritoneal dialysis (CAPD), have been widely used. The numbers of patients on CAPD has been growing rapidly in Asian countries, representing about 80% of dialysis patients in Hong Kong, and 50% in Thailand in 2011. A health care reform scheme for all Thai citizens, called the “Universal Coverage” scheme (UC) was first initiated in 2002, and it has covered renal replacement therapy for CAPD-first treatment since 2008. The usage of four 2-L daily exchanges with double-bag disconnected systems has been a standard CAPD regime in Thailand.

Adequacy targets for CAPD are primarily based on the weekly clearances of urea (Kt/V) or creatinine (Crcl) which are expressed as renal Kt/V (rKt/V), peritoneal Kt/V (pKt/V), total Kt/V (tKt/V); or renal Crcl (rCrcl), peritoneal Crcl (pCrcl), and total Crcl (tCrcl) respectively. The effect of rKt/V on survival in CAPD patients has been well-documented [6-14], but the roles of pKt/V, tKt/V and tCrcl are controversial. Some studies [9,15] found that higher pKt/V and/or tKt/V were associated with longer survival times, whereas some observational studies [6-8,10-12] and randomized controlled trials [13,14] did not find such associations. Many factors may affect these outcomes, such as dialysis dosage, residual renal function, power of test, cutoff threshold used, and follow-up period.

We therefore conducted a cohort study with 3 years’ follow up to answer the following research questions: Can tKt/V, rKt/V, and tCrcl significantly predict disease prognosis in CAPD patients? If so, what are the cutoffs that can be used to predict patients’ outcomes?

## Methods

### Setting & participants

This cohort study covered patients receiving four 2-L CAPD exchanges at 82 general hospitals, belonging to the Ministry of Public Health, Thailand. Data from the National Health Security Office (NHSO) were retrieved between January 2008 and April 2011. The study was approved by the Ramathibodi Hospital Ethical Committees.

Patients aged 15 years or older were eligible if they met the following criteria: Firstly initiated CAPD and participated in the CAPD first-policy from January 2008 to April 2011, survived more than 1 month after initiating CAPD, and had at least 1tKt/V during the studied period.

Patients were ineligible if they had the following criteria: on CAPD due to acute renal failure, aged > 100 years, tKt/V < 0.5 or > 5, tCrcl < 10 or > 400 L/week/1.73 m^2^, serum albumin level < 0.3 or > 6 g/dl, hemoglobin level < 3 or > 20 g/dl, urine volume < 0 or > 4,000 ml, ultra-filtration (UF) volume < −2,000 or > 4,000 ml, systolic blood pressure (SBP) < 40 or > 300 mmHg, or diastolic blood pressure (DBP) < 10 or > 200 mmHg.

### Clinical endpoint

The primary outcome of the study was time since first initiation of CAPD therapy to death. Patients were censored if they were lost to follow up, or survived at the end of the study (May 2011). Death referred to all cause mortality and the data were validated by cross-referencing with the death certificate database from the Ministry of the Interior.

### Prognostic factors

The studied prognostic factors were renal (i.e., rKt/V and rCrcl) and peritoneal (i.e., pKt/V and pCrcl) small solute clearances. The former clearance was estimated using a ratio of concurrent urea/creatinine excretion in 24-hour urine whereas the latter clearance was estimated using 24-hour dialysate effluent. The total small solute clearance (e.g., tKt/V or tCrcl) was the summation of renal and peritoneal small solute clearance. Urea distribution volume (V) was measured using Watson's formula [16].

These small solute clearances (i.e., rKt/V, tKt/V, tCrcl) and other variables were considered in the prognostic model, which included age, gender, body mass index (BMI), serum albumin, hemoglobin, UF volume, SBP, DBP, and co-morbidities (i.e., diabetes, hypertension and/or cardio-vascular disease (CVD)). Among these variables, tKt/V, rKt/V, tCrcl, BMI, serum albumin, hemoglobin, UF volume, SBP, and DBP were considered in the analysis as time-varying covariates, whereas the rest were fixed variables.

### Statistical analyses

#### Imputation

Among 1,177 eligible patients, the data for UF volume, hemoglobin, serum albumin, rKt/V, BMI, tCrcl, and co-morbidities were missing in 0.3%, 1.1%, 3.3%, 6.5%, 11.5%, 16.7%, and 36.3% of patients, respectively. For each patient, the last observed value was carried forward to replace missing data. Then, the rest of the missing data were imputed using multivariate chain equations. A simulation-based procedure [17,18] with the assumption that data were missing at random was applied. Logistic and linear regressions were applied to predict missing data for dichotomous and continuous data, respectively. Twenty imputations were performed to allow for the uncertainty of imputed data and the summarized values were then used [19].

#### Calibration of the cutoff threshold

The received operative characteristic (ROC) curve was used to determine the cutoff of tKt/V, rKt/V, and tCrcl that could discriminate death from living patients. Each variable was fitted in the ROC model as both continuous and categorized variables, separately. For categorical variables, they were categorized according to tertile distributions. The likelihood ratio positive (LR+) and Youden’s index (i.e., highest sensitivity + specificity −1) [20] were then used to select the cutoff threshold. The performance of the cutoff thresholds suggested by the two methods were incorporated which led to the final cutoffs.

Kaplan-Meier was applied to estimate the overall death rate and probability of death at 12-, 24-, and 36- months after first-initiated CAPD. A patient was censored if s/he had one of the following events: loss to follow-up, withdrew from the CAPD program, or alive at the end of follow up period. Cox-regression models with time varying covariates were applied to assess prognostic effects by fitting equations that separately contained rKt/V and tKt/V. Prognostic scores were then calculated from the final Cox models. The ROC curve analysis was used to estimate the area under the ROC curves (AUC) between the two prognostic scores [21].

All analyses were done using STATA software version 12. The p-value of less than 0.05 was considered statistically significant.

## Results

**Figure 1:**
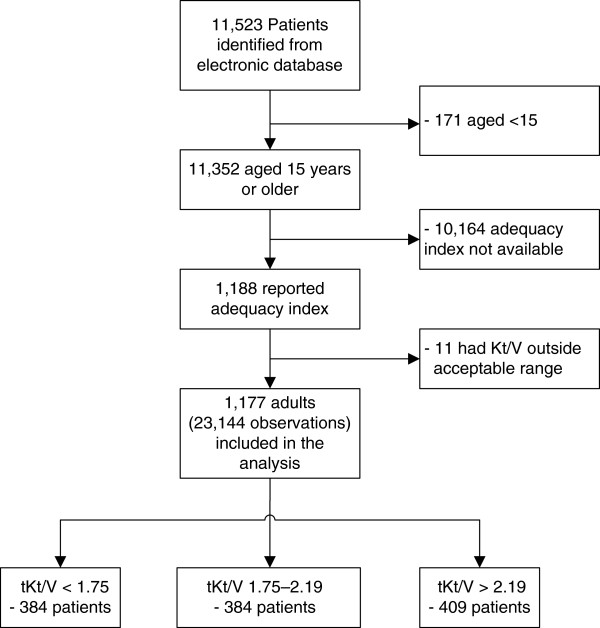
Flow of study design.

We identified 11,523 patients in the CAPD registry and follow-up databases from January 2008 to May 2011. Among them, 11,352 patients were aged 15 years or older, but only 1,188 (10.5%) patients had at least one tKt/V data. Eleven patients had a tKt/V out of the acceptable range (i.e., tKt/V < 0.5 or > 5) and thus they were excluded, leaving 1,177 patients with 23,144 observations for the primary analysis, see Figure 1. Baseline characteristics of eligible versus ineligible subjects were compared which found that eligible patients were younger (50 vs. 53, p <0.001), had slightly higher proportions of anuria (5.4% vs. 2.9%, p < 0.001), serum albumin (3.3 vs. 3.2, p < 0.001); higher mean hemoglobin (9.0 vs. 8.8, p < 0.001), urine output (704 vs. 645 ml, p < 0.001), and diastolic blood pressure (81 vs 80, p = 0.021) than ineligible patients. However, both groups had similar proportions of males (50.1% vs. 48.4%, p = 0.26, diabetes (33.2% vs. 30.5%, p = 0.12), hypertension (39.2% vs. 36.0%, p = 0.08), cardiovascular disease (4.4 vs. 3.1 years, p = 0.06), and mean SBP (141 vs. 141 mmHg, p = 0.99), see Table 1.

**Table 1:** Comparisons of baseline characteristics of eligible versus ineligible patients

**Variables**	**Eligible group**		**Ineligible group**		**p-value**
	**n**	No (%)^*^	**n**	No (%)^*^	
Age, year	1,177	50 (14)	10,346	53 (15)	< 0.001
Male	1,177	590 (50.1)	10,346	5,008 (48.4)	0.26
^£^ BMI, kg/m^2^	1,037	22.4 (4.0)	5,886	22.1 (4.0)	0.09
Serum creatinine, mg/dL	1,177	6.8 (1.1)	10,346	6.8 (1.1)	0.99
Co-morbidities					
^§^ DM	749	249 (33.2)	6,806	2,076 (30.5)	0.12
^π^ HT	749	294 (39.2)	6,806	2,447 (36.0)	0.08
^¥^ CVD	749	33 (4.4)	6,806	211 (3.1)	0.06
Anuria, N (%)	1,177	63 (5.4)	10,346	311 (2.9)	< 0.001
Serum albumin, g/dL	1,138	3.3 (0.7)	8,309	3.2 (0.7)	< 0.001
Hemoglobin, g/dL	1,164	9.0 (2.0)	8,572	8.8 (1.9)	< 0.001
Urine volume, ml	1,154	705 (529)	9,234	645 (475)	< 0.001
^μ^SBP, mmHg	1,177	141 (25)	10,346	141 (26)	0.99
^¶^DBP, mmHg	1,177	81 (14)	10,346	80 (15)	0.021

*mean (SD) for continuous data, ^£^ Body mass index, ^§^ Diabetes, ^π^ Hypertension, ^¥^ Cardiovascular disease, ^μ^ Systolic blood pressure, ^¶^ Diastolic blood pressure, Conversion factors for units: serum creatinine in mg/dL to mol/L, ×88.4; serum albumin in g/dL to g/L, ×10; hemoglobin in mg/dL to g/L, ×10.

### Calibration of tKt/V, rKt/V and tCrcl cutoff

Data for the tKt/V were categorized as < 1.75 (n = 384), 1.75-2.19 (n = 384), and > 2.19 (n = 409) using a tertile distribution. Fitting a continuous tKt/V in the ROC curve analysis yielded an optimum cutoff based on Youden’s index of 1.65. Sensitivity, specificity, and LR^+^ of this cutoff were 0.40, 0.74, and 1.50, respectively. Fitting a categorical tKt/V in the ROC analysis suggested that a cutoff of 1.75 yielded sensitivity, specificity, and LR^+^ of 0.45, 0.65, and 1.31, respectively. The AUCs were similar for both variables, i.e., 0.556 (95% CI: 0.544, 0.568) vs. 0.551 (95% CI: 0.540, 0.562), respectively. The final cutoff of 1.75 was chosen because it provided higher sensitivity.

Fitting rKt/V as a continuous variable in the ROC analysis resulted in an AUC of 0.632 (95% CI: 0.620, 0.643). Applying Youden’s index suggested the cutoff of 0.15 with sensitivity, specificity, and LR^+^ of 0.45, 0.77, and 1.96, respectively. The rKt/V was categorized into 3 groups according to a tertile distribution, i.e., < 0.25, 0.25-0.49, and > 0.49; fitting this categorical variable yielded the AUC of 0.612 (95% CI: 0.602, 0.623). Although this cutoff yielded slightly inferior performance (i.e., sensitivity, specificity, and LR^+^ of 0.53, 0.66, and 1.57 respectively), this rKt/V cutoff of 0.25 was chosen for further analyses because it provided higher sensitivity.

Fitting the tCrcl variable did not discriminate well between dead and surviving patients with the AUCs of 0.519 (95% CI: 0.506, 0.533) and 0.513 (95% CI: 0.501, 0.525) for continuous and categorical variables, respectively.

### Prognostic factors of death

Among 1,177 patients, the person-time at risk was 21,831 patient-months with a median follow-up of 22.9 months (range: 1.8–43.5 months). One-hundred and eighty eight patients died which resulted in an overall death rate of 9.9 (95% CI: 8.6, 11.4) per 100 patient-years. The probabilities of death at 12-, 24- and 36-months were 6.7% (95% CI: 5.3%, 8.4%), 19.4% (95% CI: 16.7%, 22.4%) and 29.2% (95% CI: 24.7%, 34.4%), respectively.

**Figure 2:**
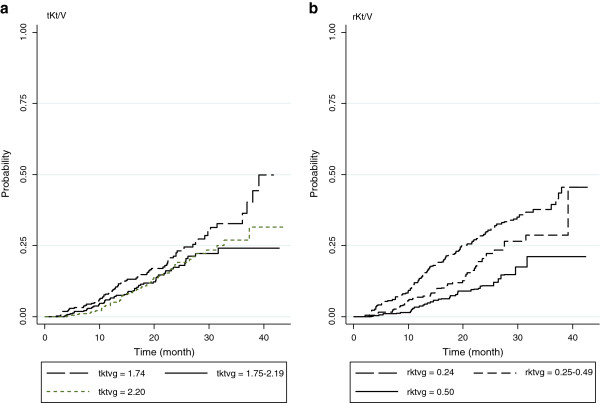
Kaplan Meier curves of death by tKt/V, and rKt/V.

Kaplan-Meier curves were plotted by tKt/V groups (see Figure 2a). This suggested that the failure curves of tKt/V of 1.75–2.19 and > 2.19 groups were similar, but they were lower when compared with tKt/V < 1.75. The overall death rates were 1.03 (95% CI: 0.83, 1.29), 0.72 (95% CI: 0.56, 0.94), and 0.71 (95% CI: 0.54, 0.92) per 100 patient-months for tKt/V < 1.75, 1.75-2.19, and > 2.19 respectively, see Table 2. The unadjusted hazard ratios (HRs) were 0.70 (95% CI: 0.49, 0.98), and 0.70 (95% CI: 0.50, 0.99) for tKt/V 1.75–2.19, and > 2.19 when compared to tKt/V < 1.75, respectively. These two tKt/V groups were then combined because their HRs were similar.

**Table 2:** Described death rates and HR according to prognostic factors: a univariate analysis

**Variables**	**Rate/100 patient-months (95% CI**^**θ**^**)**	**Unadjusted HR**^**ᴚ **^**(95% CI)**	**p-value**
tKt/V			
< 1.75	1.03 (0.83, 1.29)	1	
1.75–2.19	0.72 (0.56, 0.94)	0.70 (0.49, 0.98)	0.04
> 2.19	0.71 (0.54, 0.92)	0.70 (0.50, 0.99)	0.04
rKt/V			
< 0.25	1.65 (1.35, 2.01)	1	
0.25–0.49	1.06 (0.80, 1.40)	0.64 (0.45, 0.91)	0.01
> 0.49	0.59 (0.41, 0.85)	0.36 (0.23, 0.54)	< 0.001
tCrcl, ml/week/1.73 m^2^			
< 50	0.88 (0.66, 1.16)	1	
50–64	0.66 (0.50, 0.87)	0.74 (0.50, 1.10)	0.14
> 64	0.78 (0.59, 1.03)	0.93 (0.63, 1.39)	0.73
Age, years			
< 45	0.52 (0.39, 0.70)	1	
45–54	0.81 (0.61, 1.07)	1.64 (1.09, 2.47)	0.02
> 54	1.12 (0.92, 1.37)	2.41 (1.68, 3.44)	< 0.001
Gender			
Female	0.83 (0.68, 1.01)	1	
Male	0.81 (0.66, 0.99)	0.96 (0.72, 1.28)	0.80
^£^ BMI, kg/m^2^			
< 21.0	0.91 (0.71, 1.16)	1	
21.0 – 23.9	0.77 (0.58, 1.01)	0.78 (0.54, 1.13)	0.19
> 23.9	0.77 (0.59, 1.01)	0.71 (0.54, 1.11)	0.16
Serum albumin, g/dL			
< 3.0	1.72 (1.41, 2.10)	1	
3.0–3.4	0.76 (0.57, 0.99)	0.41 (0.29, 0.57)	< 0.001
> 3.4	0.37 (0.27, 0.50)	0.20 (0.13, 0.29)	< 0.001
Hemoglobin, g/dL			
< 9.0	1.08 (0.86, 1.36)	1	
9.0–10.9	0.84 (0.68, 1.05)	0.67 (0.48, 0.92)	0.01
> 10.9	0.49 (0.35, 0.68)	0.38 (0.25, 0.57)	< 0.001
^§^ UF volume, ml			
< 500	0.89 (0.66, 1.18)	1	
500–999	0.93 (0.75, 1.15)	0.93 (0.65, 1.33)	0.69
> 999	0.64 (0.50, 0.84)	0.59 (0.40, 0.87)	0.01
^μ^ SBP, mmHg			
< 140	0.91 (0.75, 1.11)	1	
≥ 140	0.74 (0.60, 0.90)	0.83 (0.62, 1.10)	0.19
^¶^ DBP, mmHg			
< 90	0.85 (0.71, 1.01)	1	
≥ 90	0.76 (0.59, 98)	0.92 (0.67, 1.24)	0.57
Diabetes			
Yes	1.19 (0.95, 1.49)	1.74 (1.27, 2.38)	0.001
No	0.67 (0.56, 0.81)	1	
Hypertension			
Yes	0.85 (0.69, 1.04)	1.06 (0.75, 1.49)	0.74
No	0.79 (0.65, 0.97)	1	
^¥^ CVD			
Yes	0.82 (0.49, 1.38)	0.92 (0.48, 1.78)	0.81
No	0.82 (0.71, 0.95)	1	

^θ^ Confidence Interval, ^ᴚ ^ Hazards Ratio ^£^ Body mass index, ^§^ Ultra-filtration, ^μ^ Systolic blood pressure, ^¶^ Diastolic blood pressure, ^¥^ Cardiovascular disease, Conversion factors for units: serum albumin in g/dL to g/L, ×10; hemoglobin in mg/dL to g/L, ×10.

The estimated probability of death was decreased as the rKt/V increased (see Figure 2b). The estimated death rates were 1.65 (95% CI: 1.35, 2.01), 1.06 (95% CI: 0.80, 1.40), and 0.59 (95% CI: 0.41, 0.85) per 100 patient-months for rKt/V of < 0.25, 0.25-0.49, and > 0.49, respectively. The unadjusted HRs were 0.64 (95% CI: 0.45, 0.91), and 0.36 (95% CI: 0.23, 0.54) for the groups with rKt/V of 0.25-0.49 and > 0.49 when compared with the reference group of < 0.25, respectively. Other covariables with p-value < 0.25 in the univariate analysis were age, BMI, serum albumin, hemoglobin, UF volume, SBP, and diabetes (see Table 2).

After adjusting for covariables, patients with tKt/V 1.75 or higher had 29% (HR = 0.71 (95% CI: 0.52, 0.98) significantly lower risk of death than patients with tKt/V < 1.75 (see Table 3). The effects of rKt/V on death were stronger than tKt/V, with HRs of 0.56 (95% CI: 0.38, 0.80) and 0.30 (95% CI: 0.19, 0.47) for rKt/V 0.25-0.49 and > 0.49, respectively. This suggested higher rKt/V gave lower risk of death, i.e., patients with rKt/V 0.25-0.49 and > 0.49 had approximately 44% and 70% lower risk of death when compared to patients with rKt/V lower than 0.25. We further created predictions scores from model 1 and model 2 (see Table 3). Fitting these scores in the ROC curve analysis yielded the AUCs of 0.726 (95% CI: 0.716, 0.736) and 0.753 (95% CI: 0.743, 0.763) for model 1 and model 2, respectively; indicating rKt/V was a better prognostic factor of death than tKt/V.

**Table 3:** Cox proportional hazards model for all-cause mortalities using tKt/V and rKt/V as prognostic factors

	**Model 1: tKt/V**				**Model 2: rKt/V**			
**Variables**	**Coefficient**	**SE**^**Ӕ **^	**HR**^**ᴚ **^**(95% CI**^**θ**^**)**	**p–value**	**Coefficient**	**SE**	**HR (95% CI)**	**p–value**
tKt/V								
< 1.75			1					
≥ 1.75	−0.34	0.11	0.71 (0.52, 0.98)	0.04				
rKt/V								
< 0.25							1	
0.25-0.49					−0.59	0.11	0.56 (0.38, 0.80)	0.00
> 0.49					−1.22	0.07	0.30 (0.19, 0.47)	< 0.001
Age, year								
< 45			1				1	
45 – 54	0.59	0.44	1.81 (1.12, 2.92)	0.02	0.64	0.46	1.89 (1.17, 3.04)	0.01
> 54	0.89	0.51	2.44 (1.61, 3.69)	< 0.001	0.99	0.59	2.70 (1.77, 4.13)	< 0.001
^£^BMI, kg/m^2^								
< 21			1				1	
≥ 21	−0.37	0.12	0.69 (0.50, 0.96)	0.03	−0.30	0.13	0.73 (0.53, 1.04)	0.08
Serum albumin, g/dL								
< 3.0			1				1	
3.0–3.4	−0.74	0.09	0.48 (0.33, 0.68)	< 0.001	−0.73	0.09	0.48 (0.34, 0.70)	< 0.001
> 3.4	−1.42	0.05	0.24 (0.16, 0.37)	< 0.001	−1.39	0.06	0.25 (0.16, 0.40)	< 0.001
Hemoglobin, g/dL								
< 9.0			1				1	
9.0–10.9	−0.36	0.12	0.70 (0.49, 0.98)	0.04	−0.39	0.12	0.68 (0.47, 0.97)	0.03
> 10.9	−0.89	0.09	0.41 (0.26, 0.65)	< 0.001	−0.83	0.10	0.44 (0.28, 0.69)	< 0.001
^§^UF volume, ml								
< 1,000			1				1	
≥ 1,000	−0.38	0.12	0.69 (0.49, 0.96)	0.03	−0.63	0.10	0.53 (0.37, 0.76)	0.00
^μ^SBP, mmHg								
< 140			1				1	
≥ 140	−0.36	0.11	0.70 (0.51, 0.95)	0.02	−0.37	0.11	0.69 (0.51, 0.94)	0.02
Diabetes								
Yes	0.36	0.26	1.43 (1.00, 2.03)	0.05	0.36	0.28	1.43 (0.98, 2.09)	0.06
No			1				1	

^Ӕ ^ Standard Error, ^ᴚ ^ Hazards Ratio, ^θ^ Confidence Interval, ^£^ Body mass index, ^§^ Ultra-filtration, ^μ^ Systolic blood pressure, Conversion factors for units: serum albumin in g/dL to g/L, ×10; hemoglobin in mg/dL to g/L, ×10.

Age, serum albumin, hemoglobin, UF volume, and SBP were also significant prognostic factors of death in both tKt/V and rKt/V models, but BMI and diabetes were significant only in the tKt/V model. Patients aged 45–54 and 55 years or older had respectively 1.89 (95% CI: 1.17, 3.04) and 2.70 (95% CI: 1.77, 4.13) times higher risk of death than patients aged younger than 45 years. High serum albumin resulted in better prognosis with 52% (HR = 0.48, 95% CI: 0.34, 0.70), and 75% (HR = 0.25, 95% CI: 0.16, 0.40) lower risk of death for serum albumin 3.0–3.4 g/dl and > 3.4 g/dl respectively, when compared to albumin below 3.0 g/dl.

Hemoglobin showed an inverse dose–response relationship with death; those with hemoglobin levels of 9.0-10.9 and > 10.9 g/dl had 32% (HR = 0.68, 95% CI: 0.47, 0.97) and 56% (HR = 0.44, 95% CI: 0.28, 0.69) respectively significantly lower risk of death than those with a hemoglobin level below 9.0 g/dl. UF volume 1,000 ml per day or higher reduced the risk of death by 47% (HR = 0.53, 95% CI: 0.37, 0.76). Patients whose SBP were 140 mmHg or higher had 31% (HR = 0.69, 95% CI: 0.51, 0.94) significantly lower risk of death than those whose SBP were below 140 mmHg.

Higher BMI were associated with lower mortality with the adjusted HR of 0.73 (95% CI: 0.53, 1.04) for BMI 21 kg/m^2^ or higher when compared with BMI less than 21 kg/m^2^, but this was not statistically significant. The presence of diabetes increased the risk of death by 43% with the adjusted HR of 1.43 (95% CI: 0.98, 2.09), but this was also not statistically significant.

After adjusting for covariables, patients with tCrcl ≥ 50 L/wk/m^2^ had 20% (HR = 0.80, 95% CI: 0.56, 1.13) lower risk of death than patients with tCrcl < 50 L/wk/m^2^; although this was not statistically significant, data have not been shown.

## Discussion

We conducted a retrospective cohort study of first-initiated CAPD patients with a median follow-up time of 22.9 months. The 12-, 24- and 36-months probabilities of death were 6.7% (95% CI: 5.3%, 8.4%), 19.4% (95% CI: 16.7%, 22.4%), and 29.2% (95% CI: 24.7%, 34.4%), respectively with the overall death rate of 9.9 (95% CI: 8.6, 11.4) per 100 patient-years. Prognostic factors of death were explored suggesting that rKt/V was the strongest predictor of death followed by tKt/V. The cutoff thresholds of these parameters that discriminated poor from good prognosis were ≥ 0.25 for rKt/V and ≥ 1.75 for tKt/V. Patients with the rKt/V and tKt/V higher than the cutoffs had respectively 65% and 29% lower risk of death than patients with lower levels than the cutoffs.

The mortality rate for our cohort was quite low (i.e., 6.7% and 19.4% for 1- and 2-year probabilities of death) when compared with the mortality rates in other Asian countries, e.g., Indian (20% and 40% ) [22], and China (10% and 21%) [23]. This might be explained by our study included patients who were firstly initiated CAPD while those studies included both prevalent and incident CAPD cases. Modalities of CAPD exchanges were different, we used four 2-L CAPD exchanges whereas those studied used three 2-L CAPD exchanges. Although our study had higher prevalence of diabetes compared with the Chinese study [19], our patients were younger (mean age 50 vs. 54 years) and about three times lower CVD (4.4% vs. 14.2%) than the Chinese study. We included only patients who had tKt/V data and survived at least 1 month after first-initiated CAPD. As such, our mortality rate was lower than previous studies.

Unlike rKt/V, the roles of tKt/V on mortality has not been consistently demonstrated by previous studies [6,7,12-14,20-24]. Although there was a trend in favor of higher tKt/V, those studies could not demonstrate statistical significance [6,7,12-14,24], but our study had sufficient power to detect this association. However, the prognostic effect of tKt/V needs to be confirmed by further studies since its effect was borderline significant.

A target small solute clearance has long been a critical issue. Since increasing the peritoneal dialysis prescription may increase the patients' discomfort, decrease the quality of life, and harm by metabolic glucose load, the advantages from increased dialysis dose and potential harms should be well-balanced. Unfortunately, the optimum target clearance has not been well-formulated due to lack of previous evidence and our study should be able to fill information for this issue. Both continuous and categorical variables for small solute clearances were explored in the ROC curve analyses and suggested the optimum cutoffs for tKt/V and rKt/V. These cutoffs were similar to the current tKt/V target suggested by the Kidney Disease Outcome Quality Initiative (KDOQI) guideline [25]. Two randomized controlled trials [13,14] have assessed the effects of tKt/V targets advocated by the previous KDOQI guideline. The ADEMEX study [13] compared the modified prescriptions to achieve a target tCrcl of > 60 L/week/1.73 m^2^ with the four 2-L daily exchanges of standard PD solution. They found no significant differences in patient survival, technique survival, and quality of life between intervention and control groups. The Hong Kong study [14] which had randomized patients to 3 different tKt/V targets also showed negative results.

Some studies [7,11,12] considered two or more indices of small solute clearance simultaneously in the regression models. Since tKt/V was the sum of pKt/V plus rKt/V, the two variables were highly correlated with the estimated correlation coefficient of 0.388 (p-value < 0.001). Although including them simultaneously in the same model did not cause much multicolinearity as we were aware, adding tKt/V in the model which already contained rKt/V did not improve the explanation of mortality with the area under curves of 0.753 vs. 0.755 (p = 0.82).

Increased serum albumin, hemoglobin, UF volume, and SBP > 140 mmHg were also associated with a lower risk of mortality. Interestingly, we found an inverse relationship between high SBP and risk of death. This finding is consistent with recent findings in chronic kidney disease/dialysis patients which suggested that low blood pressure could be related to coexisting co-morbidities such as heart failure, autonomic neuropathy, decreased food intake, and worse nutritional status [26-29]. These explanations may partly be attributable to these paradoxical findings, but the mechanism underlying this reverse relationship is not clearly understood.

Our study has some strengths. Our study is a large CAPD cohort with a median follow up time of nearly 2 years. We properly calibrated the rKt/V and tKt/V cutoffs using ROC curve analysis. We considered study factors (i.e., rKt/V, tKt/V, and tCrcl ) and other observed prognostic factors as time-varying co-variables in the survival analyses, which should be better in explaining disease prognosis than using only single measurements. However, our study also has some limitations. First, the study was not a randomized controlled trial and thus selection and unobserved confounding biases could not be avoided. Second, patients were only eligible if they had data for tKt/V, which was about 10% of the entire cohort; so the representativeness of the whole CAPD patients was limited. Third, we might face with survival bias because of our inclusion criteria and differences of characteristics of eligible versus ineligible patients. Our results may be generalizable to patients who firstly received CAPD with stable conditions, not patients with critical conditions. The specific etiologies of death including their prognostic factors were not evaluated due to lack of information. Among eligible patients, some variables were missing and thus multiple imputation using regression analysis was applied to predict missing values. Twenty imputations were applied taking into account the between-imputation variability.

## Conclusion

The cutoffs of 0.25 and 1.75 for rKt/V and tKt/V might be targeted to prevent mortality in CAPD patients. Serum albumin, hemoglobin, SBP, and UF volume are also associated with mortality. However, our study may face with selection and other unobserved confounders. Further randomized control trial is required to confirm these cutoffs.

## Competing interests

Phisitt Vejakama is a Ph.D. student of Clinical Epidemiology, Faculty of Medicine Ramathibodi Hospital and Faculty of Graduate Studies, Mahidol University. This study is a part of his dissertation which will be applied for granting graduation. All other authors declare that there is no duality of interest associated with this manuscript.

## Authors’ contributions

AT had full access to all of the data in the study and takes responsibility for the integrity of the data and the accuracy of the data analysis. Study concept and design: PV, AT, AI, JA, Acquisition of data: PV, PD. Analysis and interpretation of data: PV, AT, AI, JA. Drafting of the manuscript: PV, AT. Critical revision of the manuscript for important intellectual content: JA, AT, AI, PD. Final approval of the version to be published: all authors.

## Pre-publication history

The pre-publication history for this paper can be accessed here:

http://www.biomedcentral.com/1471-2369/14/28/prepub
